# Takotsubo syndrome triggered by perioperative and post-resuscitation stress: Case report

**DOI:** 10.1177/2050313X261464290

**Published:** 2026-06-30

**Authors:** Roshan Tom, Sean O’Leary, Kathy Kuang, Ariadna Robledo, Beth Teegarden

**Affiliations:** 1 Department of Anesthesiology, University of Texas Medical Branch, Galveston, TX, USA; 2 John Sealy School of Medicine, University of Texas Medical Branch, Galveston, TX, USA; 3 Department of Medicine, University of Miami, FL, USA

**Keywords:** Takotsubo syndrome, perioperative period, heart arrest, catecholamines, case report, perioperative stress, post-resuscitation, catecholamine surge, vasopressor use

## Abstract

Takotsubo syndrome or “broken heart syndrome,” is an uncommon but increasingly recognized condition that can mimic acute coronary syndrome and is characterized by transient regional left ventricular dysfunction, often with apical ballooning. Perioperative physiological stress, catecholamine surges, and vasopressor exposure are recognized triggers. This case describes suspected perioperative takotsubo syndrome in a high-risk surgical patient and emphasizes the need for vigilance and early recognition. A 66-year-old woman with hypertension, type B aortic dissection, prior stroke, carotid disease, hyperlipidemia, and tobacco use presented with a left intertrochanteric fracture requiring operative repair. During anesthesia induction, she developed profound hypotension and bradycardia refractory to fluids and vasopressors, resulting in procedure cancellation. Her initial echocardiogram demonstrated normal systolic function without regional wall motion abnormalities. Surgery was rescheduled the following day; however, she experienced pulseless electrical activity arrest postoperatively. Advanced cardiovascular life support with epinephrine achieved return of spontaneous circulation. A post-arrest echocardiogram demonstrated new mid-apical hypokinesis with an ejection fraction of 35%–40%, most consistent with takotsubo syndrome in the clinical context. She was treated with guideline-directed medical therapy, including beta-blockade and an ACE inhibitor, with subsequent stabilization and discharge. Formal coronary angiography was not performed, and the patient was lost to cardiology follow-up, limiting definitive exclusion of obstructive coronary artery disease and confirmation of left ventricular recovery. This case illustrates the multifactorial perioperative contributors to takotsubo syndrome and highlights the importance of recognizing warning features, including recurrent hypotension, catecholamine exposure, labile hemodynamics, and high-risk cardiovascular comorbidity.

## Introduction

Takotsubo syndrome (TTS), commonly referred to as broken heart syndrome, is a rare but increasingly recognized condition characterized by transient left ventricular apical ballooning.^1^ This disorder closely mimics acute coronary syndrome (ACS), often making initial diagnosis challenging. The hallmark feature is a regional wall motion abnormality leading to temporary ventricular dysfunction that often extends beyond a single coronary vascular territory and occurs in the absence of a culprit obstructive coronary lesion, although coronary artery disease may coexist and should be evaluated when clinically appropriate.^2^ The syndrome was first described in Japan, with a name that derives its meaning from the Japanese word “Takotsubo,” meaning “octopus trap,” due to the similar shape of the left ventricle during systole.

TTS is typically triggered by physical or emotional stressors, including acute infections, sepsis, medications, surgical procedures, resuscitation, or intense emotional experiences.^3^ These triggers precipitate a catecholamine surge, which is believed to contribute to myocardial stunning. Importantly, emerging registry data suggest that trigger type has prognostic significance, with physically triggered TTS associated with worse short- and long-term outcomes compared with emotionally triggered TTS.^4^ This distinction is particularly relevant in perioperative and post-resuscitation settings, where procedural stress, hemodynamic instability, and exogenous catecholamine exposure may converge. Although the exact pathophysiology remains under investigation, most patients experience complete resolution of left ventricular dysfunction within 4–6 weeks.^5^

Epidemiological studies indicate that TTS predominantly affects postmenopausal women, accounting for approximately 90% of reported cases,^5,6^ and constitutes 1%–3% of ACS cases^7^ and 0.5%–0.9% of ST-segment elevation myocardial infarctions.^6^ This report presents the clinical course of a postmenopausal female who developed TTS in the postoperative post-resuscitation setting. Written authorization compliant with the Health Insurance Portability and Accountability Act was obtained from the patient for publication of this case.

## Case description

A 66-year-old female presented for a left intertrochanteric fracture of the proximal femur, secondary to a mechanical fall. Her history is significant for hypertension, aortic dissection (Stanford type B), hyperlipidemia, osteoarthritis, hepatic hemangioma, tobacco abuse, a right middle cerebral artery (MCA) stroke with occlusion of the cervical right internal carotid artery and left carotid stenosis, and residual left-sided deficits requiring the use of a wheelchair.

**Figure 1.**
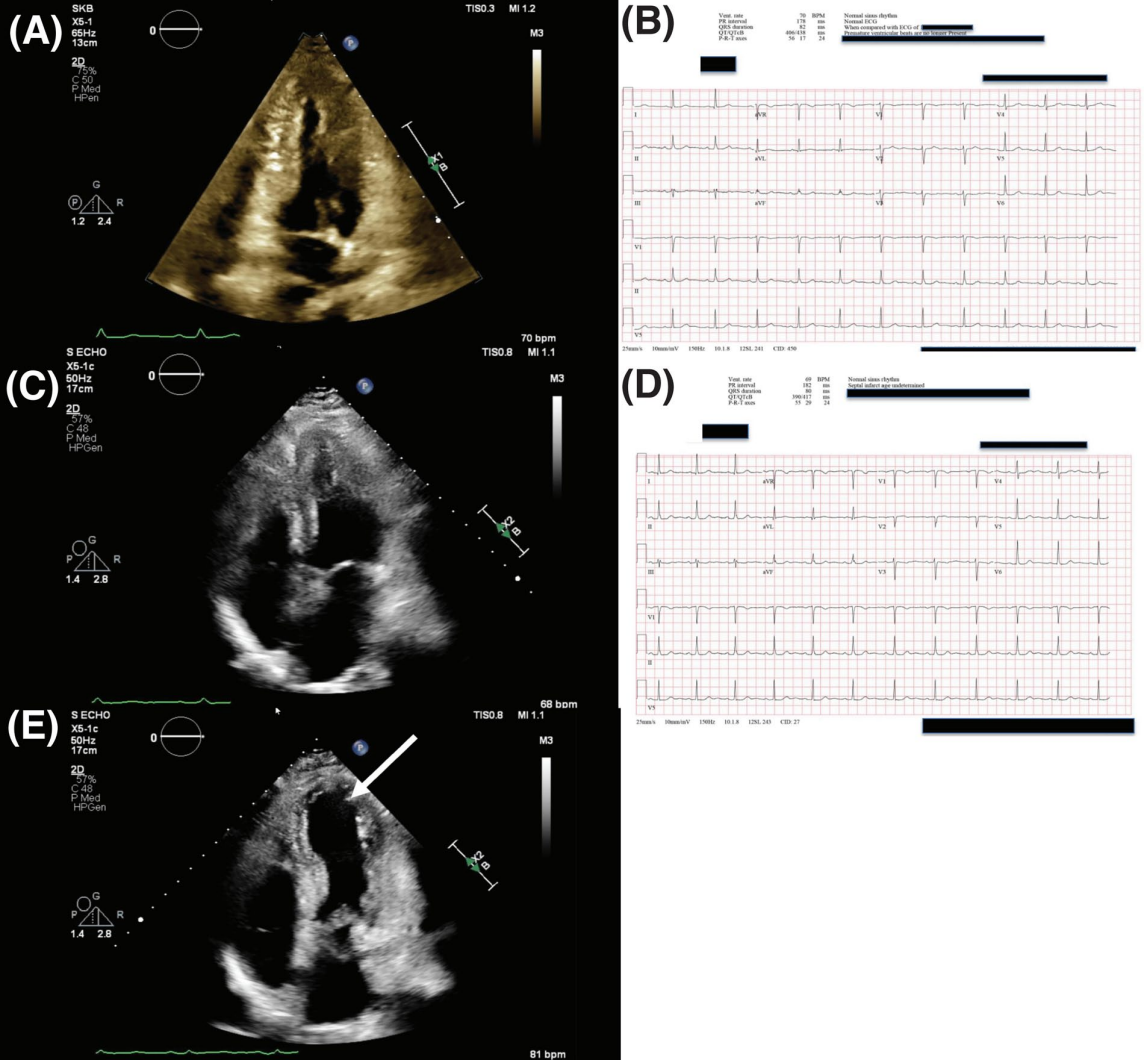
(a) Preoperative TTE demonstrating a normal EF of 60%–65% with no evidence of regional wall motion abnormalities. (b) Preoperative ECG showing normal sinus rhythm without ischemic changes or conduction abnormalities. (c) Repeat TTE following aborted surgery and perioperative hypotensive episode, confirming preserved left ventricular function and EF comparable to baseline. (d) Post-resuscitation ECG demonstrating normal sinus rhythm with an undetermined septal infarct pattern. (e) Post-resuscitation TTE demonstrating new mid-to-apical left ventricular hypokinesis with relative basal sparing and an EF of 35%–40%, consistent with a takotsubo-type stress cardiomyopathy pattern. The white arrow highlights the region of mid-to-apical left ventricular wall-motion abnormality. TTE: transthoracic echocardiography; EF: ejection fraction; ECG: electrocardiogram.

Preoperative transthoracic echocardiography (TTE) and electrocardiogram (EKG) revealed a normal ejection fraction (EF) of 60%–65% with no regional wall motion abnormalities (Figure 1(a)) and normal sinus rhythm (Figure 1(b)). During anesthesia induction with midazolam 4 mg, fentanyl 50 µg, and propofol 200 mg, the patient experienced profound hypotension and bradycardia, with mean arterial pressures (MAPs) dropping into the low 40s for several minutes. Resuscitation included fluid administration and vasopressor support with phenylephrine 300 µg, ephedrine 20 mg, and norepinephrine 197 µg. The initial vasopressor requirement may have reflected vasodilation secondary to anesthetic agents compounded by relative hypovolemia; however, this episode also represented an early period of severe perioperative hemodynamic instability and catecholamine exposure. Due to these events, the surgical procedure was aborted. The patient was extubated, noted to be at her neurological baseline, and transferred to the ICU for close monitoring.

In the ICU, the patient expressed significant anxiety regarding the events in the operating room. A repeat TTE confirmed a normal EF (Figure 1(c)), consistent with preoperative findings, and an asymptomatic elevation in troponin levels (0.066 ng/mL). The troponin elevation was attributed to demand ischemia secondary to hypotension, with levels subsequently downtrending. Overnight, her MAPs repeatedly dropped to the 50s, though they recovered with fluid administration.

The following day, the patient successfully underwent intramedullary pinning under general endotracheal anesthesia. Intraoperatively, her MAPs ranged 40–125, requiring norepinephrine and 1 L of crystalloid. During emergence, despite initial displays of strength, coughing, and spontaneous movement, she became suddenly unresponsive. Advanced cardiovascular life support (ACLS) was initiated, and naloxone was administered as she was not triggering on the ventilator. Epinephrine (1 mg) was given, resulting in the return of spontaneous circulation after 3 min. The patient was transferred back to the ICU, intubated with labile blood pressure, requiring norepinephrine and vasopressin infusions to maintain MAPs > 65 mmHg. She was extubated later that day as her clinical condition began to improve.

A complete TTE revealed new hypokinesis involving the mid-to-apical left ventricular segments with relative preservation of basal contraction and a reduced EF of 35%–40%, representing an interval change from the previously normal preoperative and repeat echocardiograms. This distribution was suggestive of a takotsubo-type wall motion pattern in the clinical context, although ischemic injury could not be definitively excluded without coronary angiography (Figure 1(d)). The EKG showed normal sinus rhythm with evidence of an undetermined septal infarct pattern (Figure 1(e)). Troponin levels peaked at 0.085 ng/mL and subsequently downtrended. The patient did not experience any new neurological deficits. Guideline-directed medical therapy was initiated, including beta-blockade and an ACE inhibitor. Formal coronary angiography was not performed during this hospitalization, and therefore, obstructive coronary artery disease could not be definitively excluded.

Over the next 2 days, the patient experienced intermittent hypotension, with MAPs to the 50s, responsive to fluids. Her recovery progressed without further complications. By the third day, with no further hypotensive episodes, she was transferred to the surgical floor. She was discharged on the seventh day to a skilled nursing facility.

The patient was instructed to follow-up with cardiology after discharge for continued management, repeat echocardiographic evaluation, and further ischemic workup, including consideration of left heart catheterization to exclude obstructive coronary artery disease. However, she was lost to follow-up. Consequently, interval recovery of left ventricular systolic function, a hallmark feature of TTS, could not be confirmed, and formal angiographic exclusion of obstructive coronary artery disease was not available. These factors represent important limitations of this report.

## Discussion

TTS is a form of nonischemic cardiomyopathy characterized by transient systolic dysfunction of the left ventricle.^8^ The underlying pathophysiology of TTS is believed to involve a state of myocardial cell hypercontraction, triggered by elevated levels of circulating catecholamines during stress.^8^ This hypercontraction is because of the formation of hypercalcified, eosinophilic transverse bands on sarcomeres, where actin and myosin remain continuously bound to calcium, leading to prolonged contraction.^9^ Additionally, the release of reactive oxygen species contributes to widespread cardiac cell death and myocardial stunning.^9^

The incidence of postoperative TTS is estimated at one in 6700 cases, making it a rare but significant concern in surgical settings.^10^ Unlike typical ACS presentations, TTS in the perioperative setting often manifests as heart failure, cardiac arrhythmias, or even cardiac arrest,^10^ which can be observed in our case. Both minor and major surgeries have been associated with TTS, indicating that procedure severity is not the sole risk determinant.^11^ In addition, TTS has been documented in both sexes following resuscitation.^12^ Among various surgeries, cesarean sections are most frequently linked to TTS, followed by orthopedic procedures.^13^ Excessive endogenous catecholamine release, combined with vasopressor use and surgical stress, is hypothesized to trigger TTS.

In the present case, the diagnosis is best interpreted as suspected TTS rather than definitively confirmed TTS. The patient had no prior documented left heart catheterization, and formal angiographic exclusion of obstructive coronary artery disease was not available during the hospitalization. This is particularly relevant given her history of right MCA infarct, cervical right internal carotid artery occlusion, left carotid stenosis, hypertension, hyperlipidemia, and tobacco use, which suggest generalized atherosclerotic disease and raise the possibility of underlying coronary artery disease. However, her preoperative TTE demonstrated preserved systolic function with an EF of 60%–65% and no regional wall motion abnormalities, which is not consistent with chronic ischemic cardiomyopathy. The development of new mid-apical hypokinesis after major perioperative physiologic stress, vasopressor exposure, and cardiac arrest remains most consistent with TTS in the clinical context, although obstructive coronary artery disease and other perioperative causes of acute myocardial dysfunction cannot be fully excluded.

Important alternative diagnoses include type 2 myocardial infarction or demand ischemia secondary to profound perioperative hypotension, obstructive coronary artery disease causing acute ischemic cardiomyopathy, perioperative stress cardiomyopathy from an alternative mechanism, pulmonary embolism with right heart strain and secondary hemodynamic compromise, and post-cardiac arrest myocardial dysfunction or myocardial stunning. The modest troponin elevation, new mid-apical hypokinesis with relative basal sparing, and temporal relationship to repeated perioperative stressors support TTS as the favored diagnosis. From an imaging standpoint, the regional dysfunction appeared more consistent with a stress cardiomyopathy pattern than a focal ischemic injury, as the abnormality involved mid-apical segments rather than a clearly isolated single-coronary-territory distribution. However, because obstructive coronary disease can coexist with TTS and coronary angiography was not performed, ischemic injury could not be definitively excluded. The lack of follow-up echocardiography confirming recovery further limits diagnostic certainty.

TTS is strongly associated with endogenous catecholamine release, which is significantly heightened during extreme stress states such as surgery, hemodynamic instability, and cardiac resuscitation.^6^ Elevated catecholamines, particularly epinephrine, are thought to contribute to myocardial stunning, microvascular dysfunction, and transient ventricular dysfunction characteristic of TTS. A meta-analysis by Nazir et al. demonstrated a significant association between epinephrine administration during cardiopulmonary resuscitation and the subsequent development of TTS.^14^ However, contemporary evidence suggests that TTS likely represents a heterogeneous syndrome in which differing trigger types may produce distinct clinical phenotypes and outcomes. Registry data have demonstrated that physically triggered TTS, including cases associated with surgery, acute illness, or resuscitation, is associated with greater clinical severity and worse short- and long-term outcomes compared with emotionally triggered TTS.^4^ In the multicenter GEIST Registry, patients with physical triggers exhibited significantly higher rates of cardiogenic shock, mechanical ventilation, in-hospital mortality, and long-term mortality than those with emotional triggers, supporting the concept that trigger type represents an important prognostic determinant in TTS.^4^ These observations suggest that the pathophysiology of perioperative and post-resuscitation TTS may involve a complex interaction between endogenous catecholamine excess, exogenous vasopressor exposure, systemic inflammation, autonomic dysregulation, and underlying physiologic vulnerability rather than a singular mechanism alone. In this context, evolving pathophysiological models emphasize that catecholamine-mediated myocardial stunning may coexist with inflammatory activation, oxidative stress, endothelial and microvascular dysfunction, and broader myocardial vulnerability, helping explain why some patients develop clinically severe TTS despite eventual recovery of systolic function.^15^ Further research is needed to clarify these mechanisms and to identify strategies for risk stratification and prevention in high-risk perioperative populations.

In this postmenopausal female patient undergoing intramedullary nailing of the femur, suspected TTS likely resulted from the convergence of multiple perioperative stressors, including sex-related susceptibility, severe anxiety after the initial aborted procedure, recurrent hemodynamic instability, vasopressor exposure, and post-resuscitation catecholamine surge. Although TTS disproportionately affects postmenopausal women, established evidence demonstrates important sex-related differences in clinical presentation, triggers, and outcomes. Men are more likely to present with physically triggered TTS, exhibit greater hemodynamic instability, and experience higher rates of cardiogenic shock, mechanical ventilation, and in-hospital mortality, whereas women more commonly present following emotional or mixed stressors.^16^ These findings further emphasize the heterogeneity of TTS phenotypes and the importance of considering both trigger type and patient sex when assessing risk and prognosis. Several warning features were present before the postoperative echocardiographic diagnosis, including the initial aborted procedure due to profound hypotension and bradycardia, substantial vasopressor requirement, mild troponin elevation, recurrent overnight hypotension, and labile intraoperative blood pressures during the subsequent procedure. The initial large vasopressor requirement on day 1 may, in part, have reflected anesthetic-induced vasodilation compounded by hypovolemia; however, it also established a pattern of significant catecholamine exposure. This exposure, combined with subsequent intraoperative hemodynamic lability, epinephrine administration during ACLS, and the physiologic stress of cardiac arrest and resuscitation, likely played a central pathophysiologic role in precipitating the observed takotsubo-like cardiomyopathy.

This case highlights the importance of recognizing the multifactorial nature of TTS, especially in perioperative and post-resuscitation settings. From a clinical standpoint, patients with multiple susceptibility factors, including postmenopausal status, significant cardiovascular or neurologic comorbidity, perioperative anxiety, and recurrent hemodynamic instability, may benefit from heightened perioperative risk stratification and multidisciplinary planning. In such patients, unexplained hypotension, labile blood pressure, troponin elevation, arrhythmia, or cardiac arrest should prompt consideration of TTS in addition to ACS, demand ischemia, pulmonary embolism, and other perioperative causes of shock. Early repeat echocardiography may be useful when clinical status changes, even if preoperative or initial postoperative imaging is reassuring. Management should focus on careful hemodynamic monitoring, correction of reversible triggers, avoidance of excessive catecholamine exposure when feasible, and individualized use of vasoactive support in collaboration with anesthesiology, cardiology, and critical care teams (Supplemental Material).

## Conclusions

This case highlights the complex interplay of perioperative and post-resuscitation factors that may contribute to suspected TTS. In this patient, anesthetic-related hypotension, possible hypovolemia, substantial vasopressor exposure, postoperative cardiac arrest, epinephrine administration during resuscitation, and underlying cardiovascular vulnerability likely converged to produce acute takotsubo-like left ventricular dysfunction. However, the absence of coronary angiography and lack of follow-up echocardiography confirming recovery limit diagnostic certainty. Clinicians should maintain a broad differential for acute perioperative myocardial dysfunction, including ACS, demand ischemia, pulmonary embolism, post-arrest myocardial stunning, and TTS. Early echocardiography, careful hemodynamic management, multidisciplinary evaluation, and follow-up imaging are important when TTS is suspected in high-risk perioperative patients.

## Supplemental Material

sj-pdf-1-sco-10.1177_2050313X261464290 – Supplemental material for Takotsubo syndrome triggered by perioperative and post-resuscitation stress: Case report Supplemental material, sj-pdf-1-sco-10.1177_2050313X261464290 for Takotsubo syndrome triggered by perioperative and post-resuscitation stress: Case report by Roshan Tom, Sean O’Leary, Kathy Kuang, Ariadna Robledo and Beth Teegarden in SAGE Open Medical Case Reports

## References

[1] Y-Hassan S De Palma R. Contemporary review on the pathogenesis of takotsubo syndrome: the heart shedding tears: norepinephrine churn and foam at the cardiac sympathetic nerve terminals. Int J Cardiol 2017; 228: 528–536. 10.1016/j.ijcard.2016.11.086 27875730

[2] Y-Hassan S Tornvall P. Epidemiology, pathogenesis, and management of takotsubo syndrome. Clin Auton Res 2018; 28: 53–65. 10.1007/s10286-017-0465-z 28917022 PMC5805795

[3] Templin C Ghadri JR Diekmann J , et al. Clinical features and outcomes of takotsubo (stress) cardiomyopathy. N Engl J Med 2015; 373: 929–938. 10.1056/NEJMoa1406761 26332547

[4] Pätz T Santoro F Cetera R , et al. Trigger-associated clinical implications and outcomes in takotsubo syndrome: results from the multicenter GEIST registry. J Am Heart Assoc 2023; 12: Article e028511. 10.1161/JAHA.122.028511 PMC10382102 37421264

[5] Ghadri J-R Wittstein IS Prasad A , et al. International expert consensus document on takotsubo syndrome (part I): clinical characteristics, diagnostic criteria, and pathophysiology. Eur Heart J 2018; 39: 2032–2046. 10.1093/eurheartj/ehy076 29850871 PMC5991216

[6] Sattar Y Siew KSW Connerney M , et al. Management of takotsubo syndrome: a comprehensive review. Cureus 2020; 12: Article e6556. 10.7759/cureus.6556 PMC6996473 32042529

[7] Matta A Delmas C Campelo-Parada F , et al. Takotsubo cardiomyopathy. Rev Cardiovasc Med 2022; 23: 38. 10.31083/j.rcm2301038 35092230

[8] Izumo M Akashi YJ. Role of echocardiography for takotsubo cardiomyopathy: clinical and prognostic implications. Cardiovasc Diagn Ther 2018; 8: 90–100. 10.21037/cdt.2017.07.03 29541614 PMC5835647

[9] Ono R Falcão LM. Takotsubo cardiomyopathy systematic review: pathophysiologic process, clinical presentation and diagnostic approach to takotsubo cardiomyopathy. Int J Cardiol 2016; 209: 196–205. 10.1016/j.ijcard.2016.02.012 26896623

[10] Hessel EA. Takotsubo cardiomyopathy and its relevance to anesthesiology: a narrative review. Can J Anaesth 2016; 63: 1059–1074. 10.1007/s12630-016-0680-4 27324891

[11] Kurisu S Inoue I Kawagoe T , et al. Tako-tsubo cardiomyopathy after successful resuscitation of out-of-hospital cardiac arrest. J Cardiovasc Med 2010; 11: 465–468. 10.2459/JCM.0b013e3283339eb9 20432510

[12] Ved YP Sharan S Bandebuche A , et al. Perioperative takotsubo stress cardiomyopathy during endoscopic spinal surgery: a case report. JBJS Case Connect 2024; 14: 12–25. 10.2106/JBJS.CC.24.00031 38848407

[13] Komamura K Fukui M Iwasaku T , et al. Takotsubo cardiomyopathy: pathophysiology, diagnosis and treatment. World J Cardiol 2014; 6: 602–609. 10.4330/wjc.v6.i7.602 25068020 PMC4110608

[14] Nazir S Lohani S Tachamo N , et al. Takotsubo cardiomyopathy associated with epinephrine use: a systematic review and meta-analysis. Int J Cardiol 2017; 229: 67–70. 10.1016/j.ijcard.2016.11.266 27889211

[15] Gragnano F Guarnaccia N Cesaro A , et al. Beyond the left ventricle: why the right heart matters in takotsubo syndrome. Int J Cardiol 2025; 428: Article 133134. 10.1016/j.ijcard.2025.133134 40058611

[16] Arcari L Núñez Gil IJ Stiermaier T , et al. Gender differences in takotsubo syndrome. J Am Coll Cardiol 2022; 79: 2085–2093. 10.1016/j.jacc.2022.03.366 35618345 PMC8972425

