# Supplementary material for: Takotsubo syndrome triggered by perioperative and post-resuscitation stress: Case report
Source: SAGE Open Med Case Rep. 2026 Jun 30;14:2050313X261464290. doi: 10.1177/2050313X261464290 (PMC13319566; doi:10.1177/2050313X261464290)
Supplement: sj-pdf-1-sco-10.1177_2050313X261464290 – Supplemental material for Takotsubo syndrome triggered by perioperative and post-resuscitation stress: Case report [file sj-pdf-1-sco-10.1177_2050313X261464290.pdf]

CARE Checklist of information to include when writing a case report

CARE case report guidelines

(cc) BY-NC-ND

Topic	Item	Checklist item description	Reported on Line
Title	1	The diagnosis or intervention of primary focus followed by the words "case report"	Title Page
Key Words	2	2 to 5 key words that identify diagnoses or interventions in this case report, including "case report"	Title Page
Abstract (no references)	3a	Introduction: What is unique about this case and what does it add to the scientific literature?	2-23
	3b	Main symptoms and/or important clinical findings	24-61
	3c	The main diagnoses, therapeutic interventions, and outcomes	24-61
	3d	Conclusion—What is the main "take-away" lesson(s) from this case?	62-108
Introduction	4	One or two paragraphs summarizing why this case is unique (**may include references**)	62-108
Patient Information	5a	De-identified patient specific information.	24-61
	5b	Primary concerns and symptoms of the patient.	24-61
	5c	Medical, family, and psycho-social history including relevant genetic information	24-61
	5d	Relevant past interventions with outcomes	24-61
Clinical Findings	6	Describe significant physical examination (PE) and important clinical findings.	24-61
Timeline	7	Historical and current information from this episode of care organized as a timeline	24-61
Diagnostic Assessment	8a	Diagnostic testing (such as PE, laboratory testing, imaging, surveys).	24-61
	8b	Diagnostic challenges (such as access to testing, financial, or cultural)	24-61
	8c	Diagnosis (including other diagnoses considered)	24-61
	8d	Prognosis (such as staging in oncology) where applicable	24-61
Therapeutic Intervention	9a	Types of therapeutic intervention (such as pharmacologic, surgical, preventive, self-care)	24-61
	9b	Administration of therapeutic intervention (such as dosage, strength, duration)	24-61
	9c	Changes in therapeutic intervention (with rationale)	24-61
Follow-up and Outcomes	10a	Clinician and patient-assessed outcomes (if available)	24-61
	10b	Important follow-up diagnostic and other test results	24-61
	10c	Intervention adherence and tolerability (How was this assessed?)	24-61
	10d	Adverse and unanticipated events	24-61
Discussion	11a	A scientific discussion of the strengths AND limitations associated with this case report	62-108
	11b	Discussion of the relevant medical literature **with references**.	109-150
	11c	The scientific rationale for any conclusions (including assessment of possible causes)	109-150
	11d	The primary "take-away" lessons of this case report (without references) in a one paragraph conclusion	102-108
Patient Perspective	12	The patient should share their perspective in one to two paragraphs on the treatment(s) they received	24-61
Informed Consent	13	Did the patient give informed consent? Please provide if requested	Yes ☒ No ☐
